# The safety concerns regarding immune checkpoint inhibitors in liver cancer patients rising mainly from CHB

**DOI:** 10.3389/fphar.2023.1164309

**Published:** 2023-04-24

**Authors:** Shike Lou, Zhujun Cao, Wanqing Chi, Xiaoyin Wang, Mingyang Feng, Lanyi Lin, Yezhou Ding, Kehui Liu, Lihong Qu, Gangde Zhao, Shisan Bao, Hui Wang

**Affiliations:** ^1^ Department of Infectious Diseases, Ruijin Hospital, Shanghai Jiao Tong University School of Medicine, Shanghai, China; ^2^ Department of Epidemiology of Microbial Disease, Yale School of Public Health, Yale University, New Haven, CT, United States; ^3^ Department of Infectious Diseases, East Hospital, Tongji University, Shanghai, China

**Keywords:** primary liver cancer, immune checkpoint inhibitor, immunotherapy, combined therapy, immune-related adverse events

## Abstract

**Aim:** To analyze the safety of immune checkpoint inhibitors in primary liver cancer patients and to identify the risk factors for immune-related adverse events (irAEs).

**Methods:** The study enrolled 106 patients with primary liver cancer, including 81 with hepatocellular carcinoma and 25 with intrahepatic cholangiocarcinoma. We analyzed the differences between groups in irAE occurrence, including those with and without targeted drugs and those who received interventional therapy.

**Results:** The incidence of irAEs was 39%, with thyroid function, liver function, and skin events being the most common. There was no correlation among irAE incidence and the liver cancer type, stage, or severity; grade of Child–Pugh score; and Barcelona Clinical Liver Cancer classification. However, being overweight was a significant risk factor for irAEs, correlating with high body mass index. The combination of targeted drugs and/or transcatheter arterial chemoembolization therapy did not increase the incidence of irAEs.

**Conclusion:** Being overweight is a potential risk factor for irAEs in primary liver cancer patients. However, there is no correlation between irAE incidence and the liver cancer type, stage, or severity or a combination of targeted drugs or transarterial chemoembolization therapy.

## Introduction

Primary liver cancer is a prevalent cancer type globally, with China accounting for more than half of all cases and a continuing increase in morbidity rates (Chen et al., 2016). Despite efforts toward early detection, a significant proportion of liver cancer patients are diagnosed at advanced stages, leading to unacceptably poor 5-year survival rates of only 12.5% (Zeng et al., 2018). The poor prognosis is mainly due to patients presenting with metastasis at the initial diagnosis, thereby losing the chance for surgical resection or liver transplantation (Bruix and Sherman, 2011; Labib et al., 2017). While local treatments such as radiofrequency ablation (RFA) and transarterial chemoembolization (TACE) are alternative approaches, they have limited benefits (Li and Ni, 2019).

More recently, immune checkpoint inhibitors (ICIs) have emerged as a promising approach in the management of malignant tumors (Sharon et al., 2014). ICIs work by blocking pathways that lead to T-cell inactivation and promote tumor cell death. The most impressive outcomes of ICIs have been observed in melanoma and non-small-cell lung cancer (Smyth et al., 2016; Dafni et al., 2019; Wang et al., 2019; Chang et al., 2020). However, the successful outcomes of ICIs are compromised due to some serious immune-related adverse events (irAEs) in the skin and thyroid (Xu et al., 2019; Qin et al., 2020). Furthermore, there is a relatively high incidence of irAEs detected from liver cancer patients following the administration of ICIs, although the efficacy of ICIs has also been observed.

It is crucial to acknowledge that although ICIs have demonstrated great potential in treating specific types of cancers, not all patients respond to this treatment equally well (Khoja et al., 2017). Several factors such as the cancer stage and type, a patient’s overall health and age, and comorbidities can all influence their response to ICIs. Therefore, it is necessary to customize the treatment approach for each patient, taking into account the potential advantages and disadvantages of combining various treatments.

Therefore, the combination of ICIs with other anti-cancer agents or with other ICIs is being explored to improve treatment efficacy with promising results (Ruf et al., 2021). However, it remains unclear whether combining immunosuppressive therapy with tyrosine kinase inhibitors and interventional therapy is safe for primary liver cancer patients, necessitating further research in this area. As such, we conducted a retrospective study to compare the incidence of irAEs between patients with liver cancer receiving ICI monotherapy and those receiving combined immunotherapy (TACE and/or TKI).

### Patients and methods

#### Study design

This retrospective study was conducted at Ruijin Hospital, Shanghai, China, and included patients with primary liver cancer, including hepatocellular carcinoma (HCC) and intrahepatic cholangiocarcinoma (ICC), who received anti-PD-1/PD-L1 treatment between July 2019 and July 2021. The study was conducted in accordance with the Declaration of Helsinki and was approved by the Institutional Review Board and Human Ethics Committee of Ruijin Hospital. Written informed consent was waived due to the retrospective nature of the study.

#### Patients

The patients included in this retrospective study met the following inclusion criteria: 1) a diagnosis of HCC or ICC confirmed by radiological or pathological findings according to the AASLD practice guidelines (Heimbach et al., 2018); 2) received at least one dose of ICIs, including PD-1 inhibitors (sintilimab, camrelizumab, pembrolizumab, tislelizumab, toripalimab, and nivolumab) or PD-L1 inhibitors (atezolizumab). Patients were excluded if they met the following criteria: 1) a history of previous ICI treatment; 2) an active or silent malignant tumor other than HCC or ICC; 3) a previous diagnosis of autoimmune disease; 4) severe cardiovascular disease (including unstable angina pectoris); 5) a serious infection; 6) a history of allergy to related drugs; and 7) pregnancy or lactation. The study was conducted in accordance with the Declaration of Helsinki and was approved by the Institutional Review Board and Human Ethics Committee of Ruijin Hospital. Written informed consent was waived due to the retrospective nature of the study.

### Data collection

We collected the following clinical and laboratory information from the electronic health system records of Ruijin Hospital: age; sex; body mass index (BMI); etiology and severity of the liver disease; absence or presence of cirrhosis; assessment of liver cancer, including the size and number of tumors, vascular invasion, and extrahepatic metastasis on imaging; stages of Barcelona Clinical Liver Cancer (BCLC); Eastern Cooperative Oncology Group performance status (ECOG PS); and treatment information on the etiology of liver disease and HCC or ICC, including PD-1 inhibitors (sintilimab, camrelizumab, pembrolizumab, tislelizumab, toripalimab, and nivolumab), TKIs, and TACE. In addition, we collected laboratory data including alpha-fetoprotein (AFP), protein induced by vitamin K absence or antagonist-II (PIVKA-II), alanine aminotransferase (ALT), aspartate aminotransferase (AST), alkaline phosphatase (ALP), glutamyl transferase (GGT), albumin (ALB), total bilirubin (TBIL), prothrombin time (PT), blood lipid levels (triglyceride (TG), total cholesterol (TC), high-density lipoprotein (HDL), low-density lipoprotein (LDL), and glucose (GLU)), and electrolyte levels (potassium (K), sodium (Na), chloride (Cl), calcium (Ca), and phosphorus (P)). Additionally, we collected serum markers of hepatitis virus infection, including HBeAg and anti-HBS, anti-HBC, anti-HBE, anti-HCV antibodies, serum HBV DNA levels, and serum HCV RNA levels.

#### Assessment of immune-related adverse events

The assessment of immune-related adverse events was conducted using the National Cancer Institute (NCI) Common Terminology for Adverse Events (CTCAE v.4.03) and was graded at each visit. Follow-up was performed every 3 weeks after the first dose of ICI until July 2021 by two experienced infectious disease physicians. irAEs, including rash, abdominal pain, diarrhea, ocular symptoms, myocardial enzymes, thyroid function, glucocorticoid levels, ACTH hormone levels, liver function, renal function, blood glucose and lipids, and chest CT images, were observed*.*

### Statistical analysis

The statistical analysis was performed using SPSS Statistics 26. Normally, the distributed measurement data were presented as mean ± standard deviation (SD), and one-way ANOVA was used to compare multiple groups. Non-normally distributed data were described using median and quartile spacing, and the Kruskal–Wallis test was used to compare mean values between multiple groups. The frequency (composition ratio) and kappa test were used to describe classification variables. A two-sided *p*-value <0.05 was considered statistically significant.

## Results

### Patient characteristics

**FIGURE 1:**
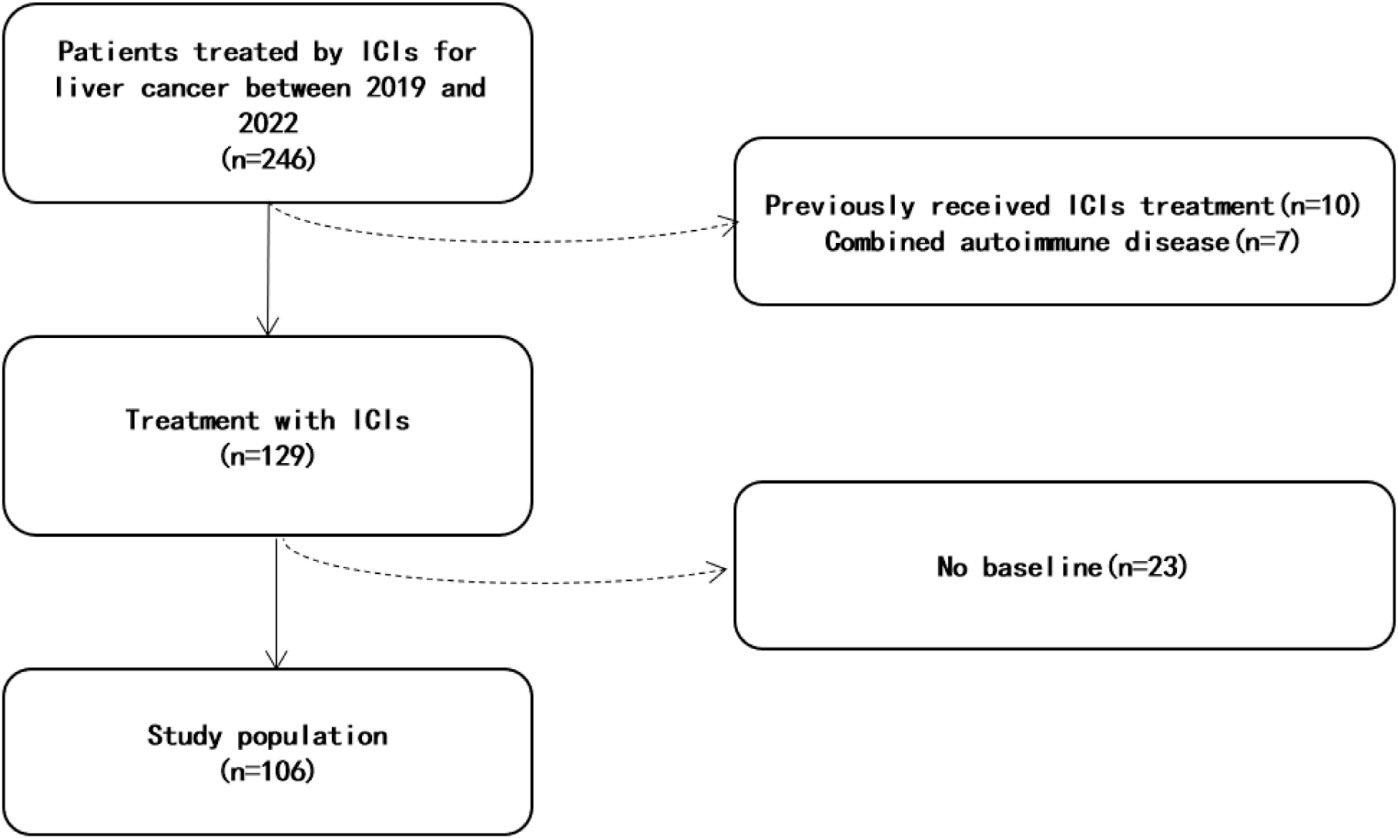
Flowchart.

This study included a total of 106 liver cancer patients treated with anti-PD-1 therapy (Figure 1). The median age was 59 years (range 27–82), and the majority of them were male (85%) (Table 1). Chronic liver disease was present in 71% of patients, with HBV being the most common etiology. Most patients (68%) had Child–Pugh grade A liver function. The majority of patients (66%) were BCLC stage C, with 13%, 16%, and 6% being stages A, B, and D, respectively. Prior to anti-PD-1 therapy, 79% of patients had received targeted therapy, 69% had undergone TACE, and 43% had liver cancer resection surgery. Only 9% of ICC patients had chemotherapy. The average number of cycles of anti-PD-1 treatment for all patients was 4.25 ± 3.80. At the final investigation, 45 patients (42%) were still receiving PD-1 treatment, while six patients died due to liver cancer progression (five) and septic shock (one).

**TABLE 1:** Baseline characteristics of patients with liver cancer treated with ICIs.

	Total (n = 106)
Age (years)	59 ± 11
Sex	
Male, n (%)	85 (80%)
Female, n (%)	21 (20%)
Underlying liver disease, n (%)	
HBV	75 (71%)
HCV	3 (3%)
Unknown	28 (27%)
Basal metabolic disease	
Hypertension	39 (37%)
Hyperlipidemia	7 (7%)
Diabetes	26 (25%)
BMI (kg/m^2^)	23 ± 3
AFP (ng/mL)	1,555 ± 5,374
CA19-9 (U/mL)	670 ± 2,246
Cirrhosis, n (%)	69 (65%)
Ascites, n (%)	27 (26%)
Vascular invasion, n (%)	42 (40%)
Extrahepatic metastasis, n (%)	48 (45%)
Postoperative recurrence, n (%)	27 (25%)
ECOG PS, n (%)	
0	101 (95%)
≥1	5 (5%)
Child–Pugh, n (%)	
A	72 (68%)
B	29 (27%)
C	1 (1%)
Unknown	4 (4%)
BCLC stage, n (%)	
**A**	13 (12%)
B	17 (16%)
C	70 (66%)
D	6 (6%)
Prior treatment, n (%)	
Surgical resection	44 (42%)
TACE	73 (69%)
TKI	84 (80%)
Chemotherapy	9 (9%)

**FIGURE 2:**
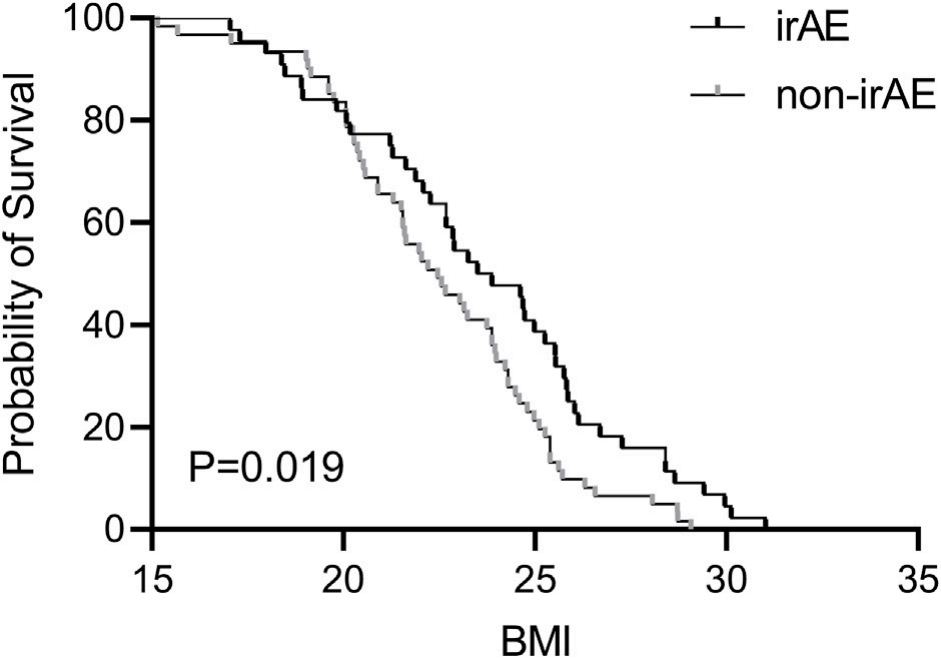
Association of irAEs with the risk factors of liver cancer patients and the Kaplan–Meier curve of BMI.

A comparison of baseline data between patients with and without irAEs (n = 44 and n = 62, respectively) was performed (Table 2). The irAE group had a BMI higher than that of the non-irAE group (23.73 *vs.* 22.55; *p* = 0.037) (Figure 2). The proportion of overweight patients (BMI ≥24 kg/m^2^) in the irAE group was higher than that in the non-irAE group (39% *vs.* 21%; *p* = 0.047). No significant differences were observed between the two groups in terms of sex, age, liver basic condition, combination of drugs, basic metabolic diseases, liver function, electrolytes, or other factors.

**TABLE 2:** Univariate analyses of the factors associated with irAEs.

	irAE (n = 44)	Non-irAE (n = 62)	*p*-value
Sex			NS
Male, n (%)	33 (75%)	52 (84%)	
Female, n (%)	11 (25%)	10 (16%)	NS
Age	58 ± 11	60 ± 11	NS
Cirrhosis, n (%)	29 (66%)	40 (65%)	NS
Basal metabolic disease, n (%)	33 (75%)	45 (73%)	NS
Extrahepatic metastasis, n (%)	23 (52%)	25 (40%)	NS
Hypertension, n (%)	17 (39%)	22 (35%)	NS
Hyperlipidemia, n (%)	4 (9%)	3 (5%)	NS
Diabetes, n (%)	10 (23%)	16 (26%)	NS
BMI (kg/m^2^)	23.7 ± 3.7	22.6 ± 3.0	0.037
Glucose (mmol/L)	5.5 ± 1.7	6.1 ± 2.4	NS
Triglyceride (mmol/L)	1.1 ± 0.3	1.0 ± 0.3	NS
Total cholesterol (mmol/L)	4.1 ± 1.0	4.2 ± 1.0	NS
Free fatty acid (mmol/L)	0.6 ± 0.3	0.6 ± 0.3	NS
PLT (10^9^/L)	139.2 ± 79.8	151.2 ± 88.7	NS
ALT (IU/L)	40.4 ± 30.q	38.0 ± 25.5	NS
AST (IU/L)	55.6 ± 44.3	57.3 ± 40.4	NS
AKP (IU/L)	171.4 ± 117.4	170.6 ± 90.2	NS
GGT (IU/L)	140.9 ± 111.4	163.4 ± 119.5	NS
TBIL (μmol/L)	20.6 ± 10.5	29.3 ± 35.6	NS
ALB (g/L)	35.3 ± 5.2	35.1 ± 5.3	NS
TBA (μmol/L)	16.6 ± 17.8	20.1 ± 28.1	NS
Na (mmol/L)	139.1 ± 3.9	138.6 ± 3.8	NS
K (mmol/L)	4.0 ± 0.5	4.0 ± 0.5	NS
Cl (mmol/L)	103.0 ± 4.5	101.5 ± 4.3	NS
CO_2_ (mmol/L)	25.8 ± 2.8	25.9 ± 2.6	NS
Ca (mmol/L)	2.2 ± 0.2	2.2 ± 0	NS
P (mmol/L)	1.1 ± 0.2	1.1 ± 0.12	NS

#### Safety of PD-1 inhibitors

**FIGURE 3:**
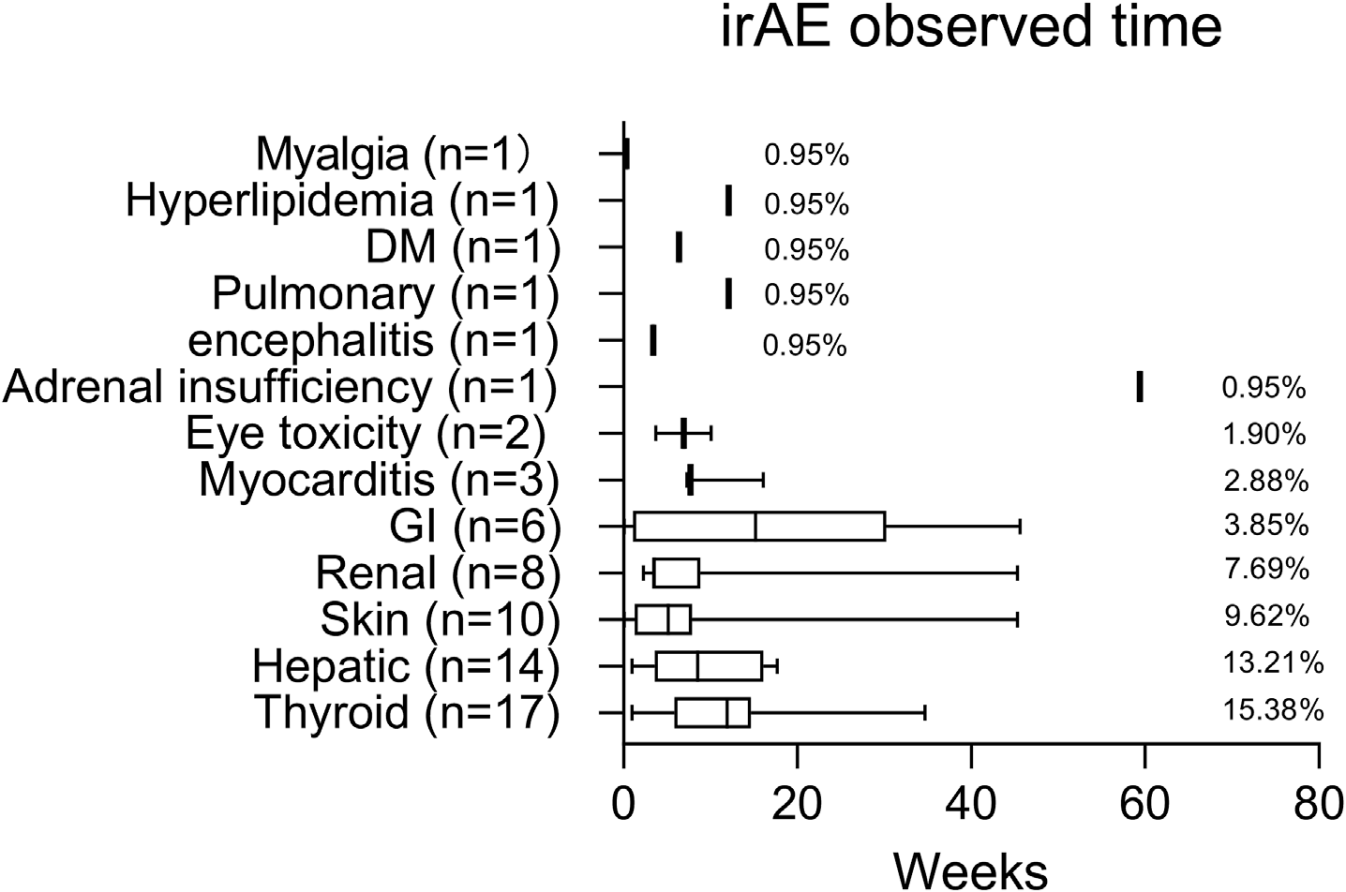
Median time (weeks) to irAE occurrence in PD-1-treated liver cancer patients.

**FIGURE 4:**
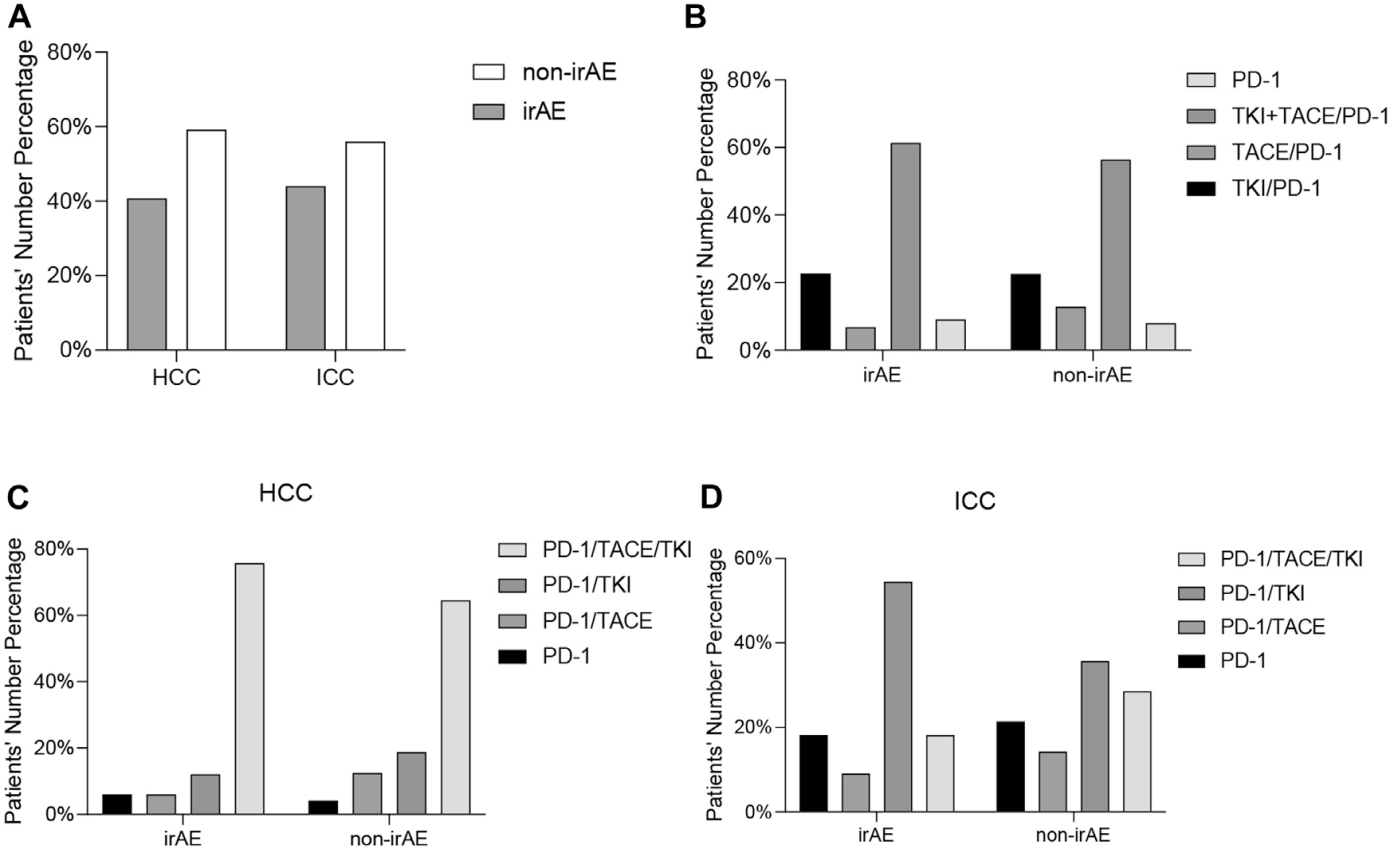
Comparison of the immune-related adverse events between HCC patients and ICC patients **(A)**. Comparison of immune-related adverse events between all patients **(B)**, HCC patients **(C)**, and ICC patients **(D)** receiving PD-1 inhibitors alone or in combination with TKI and TACE.

The safety of PD-1 inhibitors was evaluated, with a summary of the types, incidence, and average time of immune-related adverse events during ICI treatment (Figure 3). Among the 106 liver cancer patients, 44 (38%) experienced irAEs, with 14 (13%) being grade 3/4. The observed irAE subtypes were thyroid (15%), liver (13%), skin (10%), diabetes (1 patient), and hyperlipidemia (1 patient), with 15% of the patients experiencing two or more subtypes. Only one HCC patient (0.94%) experienced delayed irAEs with adrenal hypofunction 12 months after treatment discontinuation. Out of the 44 patients with irAEs, 33 (75%) were HCC patients and 11 (25%) were ICC patients (Supplementary Table S1), with no significant difference in irAE incidence between the two groups. Looking at it from another angle, 33/81 (40%) HCC patients or 11/25 (44%) ICC patients had irAEs (Figure 4). Additionally, there was no significant difference in irAE incidence among liver cancer patients who received ICI therapy alone, combined targeted drugs, or combined TACE therapy.

Regarding liver irAEs during ICI therapy, 14 (13%) patients developed full-grade hepatitis, with 10 (9%) having grade 3–4 (Table 3). There were no deaths due to irAEs. Liver adverse events were mainly increased GGT (n = 10) and ALP (n = 8), followed by TB (n = 5). Among these patients, increased AST (n = 4) and ALT (n = 2) were relatively rare. Out of the 14 patients, 11/81 (14%) were HCC patients and 3/25 (12%) were ICC patients. There were 11/78 (15%) patients with underlying hepatic diseases (HBV and HCV) and 3/28 (11%) patients without underlying hepatic diseases. The incidence of hepatic adverse events in patients with underlying hepatic diseases was slightly higher than in those without underlying hepatic diseases.

**TABLE 3:** Laboratory test with hepatic irAE patients.

Laboratory test	Baseline	irAE
ALT (IU/L)	53 ± 50	157 ± 282
AST (IU/L)	66 ± 61	140 ± 133
AKP (IU/L)	193 ± 110	285 ± 210
GGT (IU/L)	182 ± 146	261 ± 228
TBIL (μmol/L)	21 ± 13	50 ± 57
ALB (g/L)	34 ± 7	33 ± 8
TBA (μmol/L)	14 ± 11	20 ± 13

Regarding the earliest occurrence time of irAEs, we further analyzed the four groups of patients with irAEs (Table 4). There was a significant difference between PD-1 alone (23 ± 3) and PD-1/TACE (52 ± 79) groups (*p* = 0.006). PD-1/TKI was significantly different from PD-1/TACE/TKI (35 ± 32 *vs.* 95 ± 95; *p* = 0.05). Further grouping analysis showed that TKI and TACE combination treatment did not affect the earliest occurrence time of irAEs (78 ± 87 *vs.* 78 ± 83), while TACE combination treatment delayed the earliest occurrence time of irAEs (100 ± 94 *vs.* 31 ± 27; *p* < 0.0001).

**TABLE 4:** Univariate analyses of irAEs’ earliest occurrence time with Cox regression models.

Covariate	Univariate analysis (n = 44)			
	HR	95.0% CI		*p*-value
Group 1				
PD-1	Reference			
PD-1/TACE	0.099	0.019	0.513	0.006
PD-1/TKI	0.513	0.148	1.778	0.293
PD-1/TKI/TACE	0.169	0.051	0.565	0.004
Group 2				
TKI	Reference			
Non-TKI	2.410	1.124	5.165	0.095
Group 3				
TACE	Reference			
Non-TACE	0.273	0.132	0.566	<0.0001

Comparison of irAEs’ earliest occurrence time between patients receiving PD-1 inhibitors alone or in combination with TKI and TACE.

#### Subgroup analysis: patients with HBV infections

Out of the 75 liver cancer patients, 64 (85%) were HBsAg positive, with only 47 of these CHB patients having HBsAg quantification (mean value 982 ± 2213 IU/mL). Among the 71 CHB patients with HBV DNA quantification, 55 had HBV DNA levels <2 × 103 IU/mL and 16 had 2 × 103 to 2 × 107 IU/mL. Out of the 75 CHB patients, 48 (64%) had received prior antiviral therapy, 18 (24%) had simultaneous antiviral and PD-1 treatment, and 9 (12%) had PD-1 treatment only. No HBV reactivation was observed during the treatment.

The change in HBsAg levels of 31 HBsAg+ patients was collected at the end of the follow-up period, showing a ∼70% reduction from baseline to the end of the follow-up period (1,257 ± 2,606 *vs.* 504 ± 964; *p* > 0.05) (Supplementary Figure S1). These CHB patients were further categorized into three groups based on their baseline HBsAg levels (i.e., HBsAg<100 IU/mL, 100 IU/mL < HBsAg <1000 IU/mL, or HBsAg >1000 IU/mL groups). The change in HBsAg levels from baseline to the end of ICI treatment was 35 ± 26 *vs.* 36 ± 31 IU/mL (*p* > 0.05), 340 ± 297 IU/mL *vs.* 238 ± 270 IU/mL (*p* = 0.019), and 3,340 ± 3,592 IU/mL *vs.* 1,262 ± 1,328 IU/mL (*p* > 0.05) in HBsAg <100 IU/mL, 100 < HBsAg <1000 IU/mL, and HBsAg >1000 IU/mL groups, respectively.

## Discussion

Patients with HBV-associated liver tumors are at a high risk of HBV reactivation, which can result in poor overall survival outcomes (Jang, 2014). ICIs have been reported to cause HBV reactivation with varying outcomes, ranging from full recovery to liver failure. The mechanism behind HBV reactivation induced by ICIs is not fully understood, but studies have suggested that ICIs may promote Treg proliferation, disrupt immune homeostasis, or lead to the release of previously dormant viruses into circulation (Keir et al., 2008; Franceschini et al., 2009; Knolle and Thimme, 2014; Cho et al., 2017). A lack of antiviral prophylaxis has been identified as a significant factor in HBV reactivation, with some studies reporting a reactivation rate of 5.3% in HBsAg+ patients (Yoo et al., 2021). However, other studies have shown lower rates of reactivation in HBsAg+ patients (1.0%) and no reactivation in HBsAg− patients, regardless of the HBcAb status (Yoo et al., 2021).

The majority of patients in the present study were HBV positive (HBsAg^+/−^) and had received antiviral therapy before the initiation of the ICI treatment, and no HBV reactivation was detected. Moreover, concurrent antiviral therapy with ICIs did not lead to HBV reactivation, emphasizing the significance of antiviral prophylaxis in preventing HBV reactivation in immunosuppressed patients (Lee et al., 2020; Yoo et al., 2021; Zhao et al., 2022).

ICIs have been shown to potentially have antiviral effects by decreasing T-cell exhaustion and enhancing virus-specific T-cell responses in HBV infections. Studies have demonstrated sustained antiviral effects of the PD-L1 blockade in chronic hepatitis B infections (Fisicaro et al., 2010). In a pilot study of virally suppressed HBeAg− patients, the checkpoint blockade was well tolerated and led to a decline in HBsAg levels in most patients (Gane et al., 2019). These findings support the results of our current study, which showed a decrease in HBsAg levels, particularly in patients with a baseline HBsAg level >1000 IU/mL. Our data suggest that ICIs may also have a role in antiviral functions (Hagiwara et al., 2022; Pan et al., 2022).

The incidence of immune-related adverse events in most solid tumors is reported to be between 17.1% and 27%, with 4%–6% of them being grade 3/4. However, in liver tumor patients, the incidence of irAEs is higher, ranging from 42.9% to 54.6% (Sangro et al., 2020; Sonpavde et al., 2021), with a grade 3/4 incidence of 10.7% (Julien et al., 2020). Our study, consistent with previous research studies, showed an incidence of irAEs of 38.46% and a grade 3/4 incidence of 13.33%. Skin events were the most common, followed by gastrointestinal and liver events. Among the irAEs observed in our patients, the top three were thyroid, liver, and skin-related events. We also noted a rare case of hyperlipidemia, which may be a potential irAE, but further clinical studies are necessary for its validation.

The most frequently reported gastrointestinal adverse reactions to ICI treatment include decreased appetite, nausea, vomiting, diarrhea, and constipation. In our study, the incidence of gastrointestinal adverse reactions did not rank among the top few adverse reactions. This could be due to the prophylactic use of antiemetic drugs, such as serotonin receptor antagonists and hormones, prior to ICI treatment.

Hepatic irAEs typically occur between 4 and 12 weeks after ICI treatment. Among patients with non-liver tumors, the incidence of hepatic irAEs is around 4%–6%, with 1%–2% of patients experiencing grade 3/4 adverse reactions (Julien et al., 2020). However, for patients with liver tumors, the incidence and severity of hepatic irAEs are important considerations. Previous studies have reported that hepatic irAEs occur in 4.3%–24.3% of patients with liver tumors, with grade 3/4 reactions occurring in 6.5%–6.9% of cases (Chen et al., 2020; Julien et al., 2020; He et al., 2021). In our study, only 13.21% of the patients developed full-grade hepatitis, with 9.43% experiencing grade 3/4 hepatitis. These results suggest that ICI therapy for liver tumors is generally safe.

The median time for the diagnosis of delayed irAEs was 6 months, following the treatment with ICIs, within a range of 3–28 months (Couey et al., 2019). For melanoma patients treated with anti-PD-1 therapy for more than 12 months, delayed irAEs occurred in 5.3% of cases (Julien et al., 2020). In a clinical trial for advanced HCC, 15.5% of patients experienced delayed irAEs, with grade 3/4 events being observed in 5.8% of patients after 100 days of follow-up (Julien et al., 2020). To date, there have been no reports of delayed irAEs related to liver tumors in the real world. In our study, the incidence of delayed irAEs was 0.94%, providing valuable reference for evaluating the occurrence of delayed irAEs in liver tumors in the real world.

Previous studies have shown that a higher BMI increases the risk of irAEs in patients with NSCLC, melanoma, renal cell carcinoma, urothelial carcinoma, and squamous cell carcinoma of the head and neck, suggesting that BMI may contribute to irAEs (Cortellini et al., 2020; Gülave et al., 2021; Leiter et al., 2021). In our study of patients with primary liver cancer, we observed that patients with irAEs had a higher BMI compared to non-irAE patients, with overweight patients experiencing an increased incidence of irAEs. These findings suggest that an elevated BMI may be a risk factor for irAEs, possibly due to the increased PD-1/PD-L1 expression in obesity-related immune cells (Ilavská et al., 2012). However, further prospective studies are needed to investigate this association in more detail.

Immunotherapy revolutionizes the therapeutic landscape of several tumor types, playing an important role in combination therapy of patients with advanced HCC. However, only a fraction of HCC patients benefit from immunotherapy (Pinato et al., 2020). Thus, identifying reliable response predictors would improve the efficacy of immunotherapy for HCC patients (Di Federico et al., 2022). PD-L1 and tumor mutational burden (TMB), gut microbiota, and several other potential predictors for HCC are currently being evaluated (Rizzo et al., 2021b; 2022). More recently, there have been several reports that cancer patients with 1/10 tumor cells expressed PD-L1 and PD-L1 have worse outcomes (shorter recurrence-free survival (RFS) and OS following immunotherapy) (Lei et al., 2020). A high TMB is associated with higher mutation rates and formation of neoantigens and enhanced anti-cancer immune responses, improving the clinical outcomes (Samstein et al., 2019; Rizzo et al., 2021a). However, data on the role of TMB and MSI as predictive biomarkers for HCC are scarce and limited to clinical trials or case reports (André et al., 2020; Merino et al., 2020). A study suggests that there are potential predictive roles of the MMR, MSI, TMB, and PD-L1 expression detection in ICC patients (Ricci et al., 2020).

Early crossing of survival curves in randomized clinical trials (RCTs) of immune checkpoint blockers suggests excess mortality in the first month of immunotherapy (Ricci et al., 2020). However, combined therapy substantially reduced early deaths compared to single immunotherapy (Viscardi et al., 2022).

Immunotherapy remains a significant challenge for patients with liver tumors. While it has been successfully applied to HCC patients with compensated cirrhosis or Child–Pugh A status, there is limited application in those with decompensated cirrhosis (Child–Pugh score B or C) or patients who have undergone liver transplantation (Akce et al., 2022). Although the application of atezolizumab/bevacizumab as the first-line treatment for advanced HCC has significantly improved clinical outcomes, there is a lack of understanding about the appropriate second-line treatment for these patients after immunotherapy (Wong et al., 2022).

Sipuleucel-T (a cancer vaccination) is currently approved for the treatment of metastatic castration-resistant prostate cancer with promising outcomes. Such a concept could also be utilized in HCC patients in the future, in combination with immunotherapy. Additionally, more attention may be focused on immune drug resistance, which is still the main contributing factor restricting the development of ICIs (Chen et al., 2022).

There are some limitations in the current study. First, this was a single-center retrospective study, which should be confirmed in the future as prospective multicenter studies. Second, the use of different PD-1 inhibitors may have impacted the uniformity of the treatment procedures. Third, the follow-up period was relatively short, and some patients were still receiving ICI treatment at the end of the study, which may compromise the statistics. The sample size was relatively small, and the HCC patients were the residents of the eastern coast of China. We will verify such information from the HCC patients from different regions and/or countries with different genetic backgrounds in the future.

## Conclusion

This study provides important real-world evidence on the safety of combining immune checkpoint inhibitors (ICIs) with targeted drugs and interventional therapy and the risk of HBV reactivation in patients with HBV-related liver cancer. Effective antiviral prophylaxis is critical to ensuring the safety of ICI therapy in these patients. Our findings suggest that BMI may be a potential risk factor for irAEs, with overweight patients being more susceptible. While ICIs combined with targeted drugs or TACE therapy have a manageable safety profile, the occurrence of irAEs still requires close monitoring. Notably, the combined TACE treatment delayed the earliest occurrence of irAEs, and further investigation is warranted to understand the underlying mechanism.

## Data Availability

The original contributions presented in the study are included in the article/Supplementary Material; further inquiries can be directed to the corresponding authors.

## Abbreviations

HCC, hepatocellular carcinoma; ICC, intrahepatic cholangiocarcinoma; irAE, immune-related adverse reactions; TACE, transarterial chemical embolization; ICIs, immune checkpoint inhibitors; HBV, hepatitis B virus; HCV, hepatitis C virus; PD-1, programmed death-1 receptor; PD-L1, programmed death-ligand 1; CTLA-4, cytotoxic T-lymphocyte antigen 4; TKI, tyrosine kinase inhibitors; BCLC, Barcelona Clinical Liver Cancer; ALT, alanine aminotransferase; AST, aspartate aminotransferase; AKP, alkaline phosphatase; GGT, glutamyl transferase; TB, total bilirubin; BMI, body mass index.
