# Supplementary material for: The safety concerns regarding immune checkpoint inhibitors in liver cancer patients rising mainly from CHB
Source: Front Pharmacol. 2023 Apr 24;14:1164309. doi: 10.3389/fphar.2023.1164309 (PMC10165088; doi:10.3389/fphar.2023.1164309)
Supplement: Supplementary file 1 [file DataSheet1.docx]

Supplementary Material

Table S1. Baseline characteristics of patients with liver cancer treated with ICIs by type of liver cancer

|  | HCC (n=81) | ICC (n=25) |
| --- | --- | --- |
| Age (years) | 58±11 | 63±10 |
| Sex |  |  |
| Male, n (%) | 72 (89%) | 12 (48%) |
| Female, n (%) | 9 (11%) | 13 (52%) |
| Underlying liver disease, n (%) |  |  |
| HBV | 71 (88%) | 4 (16%) |
| HCV | 3 (4%) | 0 (0%) |
| Unknown | 7 (9%) | 21 (84%) |
| Basal metabolic disease |  |  |
| Hypertension | 28 (35%) | 11 (44%) |
| Hyperlipidemia | 3 (4%) | 4 (16%) |
| Diabetes | 23 (28%) | 3 (12%) |
| BMI (kg/m^2^) | 23±3 | 23±3 |
| PLT (10^9^/L) | 136±83 | 179±83 |
| ALT (IU/L) | 38±25 | 41±34 |
| AST (IU/L) | 61±45 | 41±26 |
| AKP (IU/L) | 166±94 | 188±126 |
| GGT (IU/L) | 149±107 | 169±144 |
| TBIL (μmol/L) | 27±30 | 22±20 |
| ALB (g/L) | 35±6 | 37±4 |
| TBA (μmol/L) | 20±27 | 13±12 |
| PT (sec) | 13±2 | 12±1.45 |
| AFP (ng/mL) | 1997±5972 | 3.47±2 |
| CA199 (U/mL) | 64±91 | 2881±4223 |
| Cirrhosis, n (%) | 66 (81%) | 3 (12%) |
| Ascites, n (%) | 25 (31%) | 2 (8%) |
| Vascular invasion, n (%) | 3 (45%） | 6 (24%) |
| Extrahepatic metastasis, n (%) | 37 (46%) | 11 (44 %) |
| Postoperative recurrence, n (%) | 24 (30%) | 3 (12%) |
| ECOG PS, n (%) |  |  |
| 0 | 76 (94%) | 25 (100%) |
| ≥1 | 5 (6%) | 0 (0%) |
| Child–Pugh, n (%) |  |  |
| A | 53 (65%) | 19 (76%) |
| B | 24 (30%) | 5 (20%) |
| C | 1 (1%) | 0 (0%) |
| Unknown | 3 (4%) | 1 (4%) |
| BCLC Stage, n (%) |  |  |
| A | 8 (10%) | 5 (20%) |
| B | 13(16%) | 4 (16%) |
| C | 57 (70%) | 13 (52%) |
| D | 3 (4%) | 3 (12%) |
| Prior treatment, n (%) |  |  |
| Surgical resection | 34 (42%) | 10 (40%) |
| TACE | 64 (79 %) | 9 (32%) |
| TKI | 69 (85%) | 17 (68%) |
| Chemotherapy | 0 (0%) | 9 (36%) |

Figure S1. HBsAg decline. Mean changes in serum HBsAg from baseline to the end.A-D:31 HBsAg+ patients,E-H:28 HBsAg+ patients receiving antiviral therapy
